# Lipids and Antiplatelet Therapy: Important Considerations and Future Perspectives

**DOI:** 10.3390/ijms22063180

**Published:** 2021-03-20

**Authors:** Nina Đukanović, Slobodan Obradović, Marija Zdravković, Siniša Đurašević, Maja Stojković, Tomislav Tosti, Nebojša Jasnić, Jelena Đorđević, Zoran Todorović

**Affiliations:** 1 High Medical School Milutin Milanković, Crnotravska 27, 11000 Belgrade, Serbia; djninank@gmail.com; 2 Clinic of Emergency Medicine, Military Medical Academy, University of Defence, Crnotravska 27, 11000 Belgrade, Serbia; sloba.d.obradovic@gmail.com; 3 Medical Faculty of the Military Medical Academy, University of Defence, Crnotravska 27, 11000 Belgrade, Serbia; 4 Dr Subotića 8, School of Medicine, University of Belgrade, 11000 Belgrade, Serbia; sekcija.kardioloska@gmail.com (M.Z.); maja.stojkovic@med.bg.ac.rs (M.S.); 5 Dr Žorža Matea bb, University Medical Centre “Bežanijska kosa”, 11070 Belgrade, Serbia; 6 Faculty of Biology, University of Belgrade, Studentski trg 3, 11000 Belgrade, Serbia; sine@bio.bg.ac.rs (S.Ð.); jasnicn@bio.bg.ac.rs (N.J.); jelenadj@bio.bg.ac.rs (J.Ð.); 7 Faculty of Chemistry, University of Belgrade, Studentski trg 12-16, 11000 Belgrade, Serbia; tosti@chem.bg.ac.rs

**Keywords:** P2Y12 inhibitors, discontinuation, HDL

## Abstract

Lipids play an essential role in platelet functions. It is known that polyunsaturated fatty acids play a role in increasing platelet reactivity and that the prothrombotic phenotype plays a crucial role in the occurrence of major adverse cardiovascular events. The ongoing increase in cardiovascular diseases’ incidence emphasizes the importance of research linking lipids and platelet function. In particular, the rebound phenomenon that accompanies discontinuation of clopidogrel in patients receiving dual antiplatelet therapy has been associated with changes in the lipid profile. Our many years of research underline the importance of reduced HDL values for the risk of such a rebound effect and the occurrence of thromboembolic events. Lipids are otherwise a heterogeneous group of molecules, and their signaling molecules are not deposited but formed “on-demand” in the cell. On the other hand, exosomes transmit lipid signals between cells, and the profile of such changes can be monitored by lipidomics. Changes in the lipid profile are organ-specific and may indicate new drug action targets.

## 1. Platelet Lipids

Lipids represent a large group of small organic molecules that play an essential role in maintaining cell homeostasis. As a structural constituent of a biological membrane, they play a vital role in membrane interaction curvature and fluidity. The lipids can be classified as fatty acids (FAs), prenols, sterols, glycerophospholipids, glycerolipids, sphingolipids, polyketides, and saccharolipids based on their backbone structure (Table 1).

The platelets represent tiny small colorless blood constituents that form clots and stop or prevent bleeding. There are several distinct families of lipids in platelets, such as phospholipids, sphingolipids, steroids, and prenol lipids, and fatty acid isomers with various chain lengths and saturation. Phospholipids are major structural lipids in human platelets. The phospholipids contain a hydrophilic moiety phosphoric group and fatty acid as a hydrophobic part. Those lipids arrange themselves in membranes with FAs orientated to the core and polar headgroups facing the aqueous phase.

**Figure 1:**
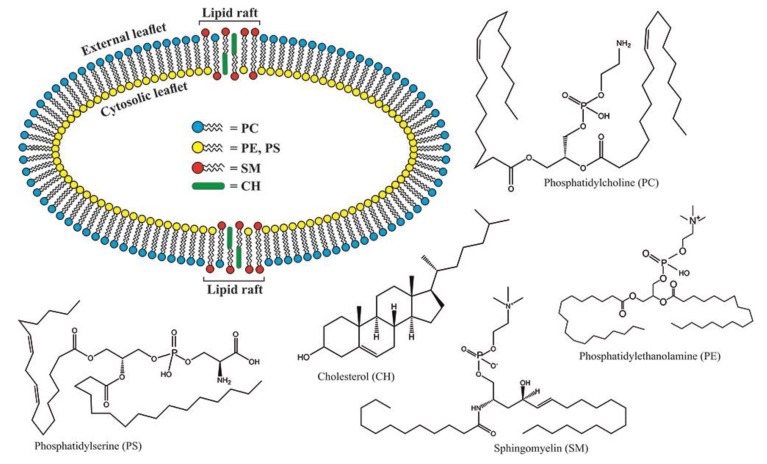
Lipid profile of platelet plasma membrane: PC—DL-a-Phosphatidylcholine, distearoyl; PE—1,2-dioleoyl-sn-glycerophosphoethanolamine; PS—1-oleoyl-2-palmitoyl-sn-glycero-3-phospho-L-serine; SM—N-Lauroyl-D-erythro-sphingosylphosphorylcholine; CH—cholesterol.

The platelet membrane structure is very complex, with a large number of lipids embedded in it (Figure 1). The most abundant are aminophospholipids (APL), such as phosphatidylcholine (PC) and sphingomyelin (SM), oriented outside in asymmetrical bilayer membrane, contrary to cytosol compounds such as phosphatidylethanolamine (PE) and phosphatidylserine (PS). The aminophospholipids’ circling through the membrane is the most critical process responsible for the activation, aging, and apoptosis of platelets [1]. It has been reported that the lack of PS on the platelet’s surface might impair their coagulation role [2,3]. Clark et al. were able to identify which platelet-specific PE/PS are more procoagulant depending on their side-chain FA composition [1]. They also reported that the same protein was essential for PE/PS externalization during thrombin activation and energy depletion but not for apoptosis. Platelet-specific APLs optimally supported tissue factor-dependent coagulation in human plasma, vs. APL with longer or shorter fatty acyl chains [4]. Van Kruchten et al. confirmed that TMEM16F (a Ca^2+^-gated ion channel required for Ca^2+^-activated PE exposure on the cell surface) is required for agonist-triggered scrambles but not for platelet aging/apoptosis [5].

Short-chain fatty acids with 14–16 carbons predominate in the plasma membrane of resting platelets: palmitic (~17%), stearic (15%), oleic (19%), and linoleic acid (11%), while arachidonic acid contributes 18% [6]. Phospholipids are mostly composed of unsaturated fatty acids (>60%), particularly of polyunsaturated fatty acids (PUFA, ~36%), and the ratio of unsaturated and saturated acids in them is 1.6.

Tang et al. thoroughly investigated how P4 ATPase can influence phospholipid translocation mechanisms. They revealed that phospholipid asymmetry was maintained by several regulatory mechanisms [7]. Their conclusion was confirmed by Kemp et al., who pointed out the importance of flippase, a transmembrane lipid transporter that belongs to the ABC-transport protein family [8].

Using the lipidomics, Clark et al. showed that two PS and five PEs shaped thrombin, collagen, or ionophore-reactivated human platelets. Those processes were controlled by calcium mobilization and protease-activated receptors. Energy depletion (aging) externalized the same APLs in a calcium-dependent manner, and all stimuli externalized oxidized phospholipids, hydroxyeicosatetraenoic acid-PEs [4]. It can be assumed that calcium mediates the binding of gamma carboxy glutamic acid residues on the amino-terminal portion of prothrombin with negatively charged phosphate groups on phospholipid micelles, facilitating the prothrombin activity [8].

The platelets respond to activation by promoting the phospholipase and releasing the arachidonic, palmitic linoleic, and stearic acids from the membrane [9,10]. Namely, oxidized lipids (oxylipins) play an important role in the regulation of platelet function. Polyunsaturated fatty acids (PUFAs)—arachidonic acid (AA), linoleic acid (LA), eicosapentaenoic acid (EPA), and others, participate in the formation of essential lipid products that play a crucial role in activating or inhibiting platelet function. The influx of calcium into platelets leads to cytosolic phospholipase A2 (cPLA2) translocation into the platelet membrane. cPLA2 separates free fatty acids from membrane phospholipid molecules, and they are further metabolized into bioactive lipid products that affect transmembrane G-protein-coupled receptors and intracellular peroxisome proliferator-activated receptors (PPARs). Oxylipins can penetrate through the cell membrane and act in other cells. Cyclooxygenases, lipoxygenases, and cytochromes P450 play a key role in their metabolism. For example, under the influence of cyclooxygenase, platelet-derived thromboxane A2 is produced from PUFA, a series of platelet-inhibiting substances (prostaglandins D1, D2, D3, E1, E3 and I3, and thromboxanes A1 and A3), as well as PGE2 with both effects. Numerous specialized pro-resolving lipid mediators (SPMs) are formed by the metabolism of arachidonic, eicosapentaenoic, and docosahexaenoic acids in the presence of cyclo- and lipoxygenases. SPMs involve lipoxins, resolvins, and others. The synthesis of resolvins D, E, and T is especially significant. Resolvin E1 (RvE1) is derived from PUFA eicosapentaenoic (EPA) and has an inhibitory effect on platelets via ChemR23 (chemerin R23) and the G protein-coupled receptor [11,12]. This inhibitory action of RvE1 refers to the activation of platelets with ADP and thromboxane B4, but not with collagen. Resolvin D1, on the other hand, has the opposite effect on ADP-induced platelet activation. Interestingly, however, resolvin D1 acted protectively in an experimental model of ischemic-reperfusion brain damage in rats, probably by inhibiting NLRP3 (NOD, LRR, and pyrin domain-containing protein 3) inflammasomes [13]. Therefore, resolvin’s effect on platelet function and the wider implications in organ-crosstalk remain to be clarified.

Valles et al. showed that during maturation, increasing concentration of saturated fatty acids 16:0 and 18:0 and 20:4, and decreasing 20:5 could be the reason for higher platelet activity [14]. Platelet-specific APLs optimally supported tissue factor-dependent coagulation in human plasma vs. APL with longer or shorter fatty acyl chains, demonstrating the importance of the fatty acids as molecular determinants of APL that regulate hemostasis.

Eicosanoid signaling plays an important role in platelet physiology and vascular disease prevention. The use of the cyclooxygenase inhibitor, such as aspirin, in the prevention of cardiovascular events, was depicted by Les et al. [15]. They investigated various factors that may affect coagulation in cardiovascular diseases, including antithrombotic agents in stroke, antiphospholipid syndrome, and other prothrombotic conditions. Arachidonic acid (AA) can be regarded as the most important ancestor of the eicosanoid made by lipoxygenases and cyclooxygenases. Robert Morin demonstrated AA’s importance, assuming that prostaglandin synthesis released arachidonic acids from platelet phospholipids. Thrombin-induced aggregation also results in the release of arachidonic acid, primarily from PC and phosphatidylinositol (PI). In addition, free AA availability may be regulated by platelet phospholipase A2 activity [16].

**Figure 2:**
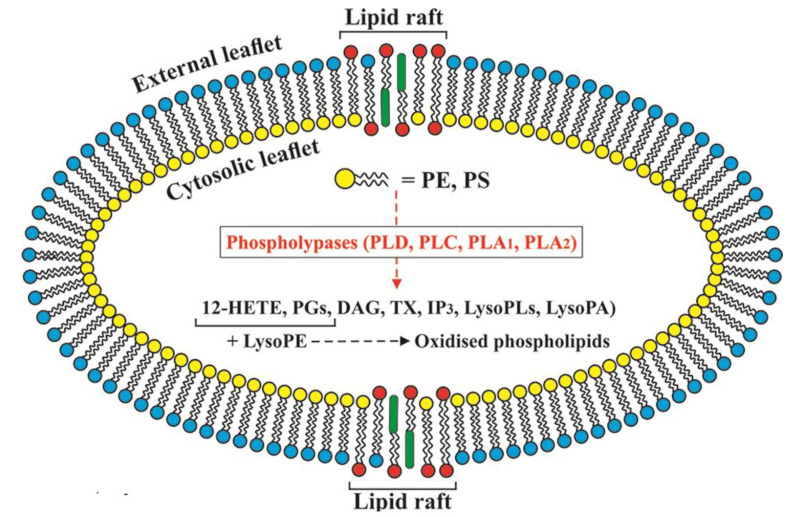
Enzymatic mechanisms that generate oxidized phospholipid during platelets activation: PLD—Phospholipase D; PLC—Phospholipase C; PLA_1_—Phospholipase A1; PLA_1_—Phospholipase A2; 12-HETE—12- hydroxyicosatetraenoic acid; PGs—Prostaglandins; DAG—Diacylglyceride; TX—Thromboxane; IP_3_—Inositol triphosphate; LysoPLs—Lysophospholipids; LysoPA—Lysophosphatidic acid; LysoPE—Lysophosphatidylethanolamine.

Thomas et al. investigated the human platelets and discovered enzymatic mechanisms that generate significant amounts of specific oxidized phospholipid molecular species during acute activation by thrombin, collagen, or Ca^2+^ ionophore (Figure 2). Based on those findings, it can be concluded that phospholipid oxidation is not an accidental consequence of inflammatory disease but a regulated process necessary during physiological hemostasis [17]. Besides oxidized phospholipids, sphingolipids and ceramides also play a vital role in membrane functions. They act as intracellular signaling messengers and take part in lipid raft-dependant signaling [18].

Lipid rafts are parts of the platelet membrane associated with the release of extracellular vesicles and that interact with the cytoskeleton. These are microdomains of the membrane rich in cholesterol and sphingolipids. There are multiple subpopulations of rafts, e.g., light, with cerebroside or ganglioside GM1, and dense, with cholesterol and flotillins [19]. In platelet rafts, more than 50 proteins were identified. Among them, alpha-, beta- and gamma-fibrins are found in activated platelet rafts, while in the resting ones, fibrinogens alpha, beta, and gamma could be found in the non-raft fractions. There are also actin, myosin, small GTPases, and regulatory proteins, such as vasodilator-stimulated phosphoprotein (VASP). Of the neutral glycophospholipids, lactosylceramide is the most abundant in the membrane’s raft zone, and ganglioside GM3 of the acidic ones. There is also much sulfatide in rafts (it has a vital role in intercellular communication), ganglioside GM1, and others [20,21].

Palmitoylation is a posttranslational modification of a protein in the raft fraction of a platelet membrane. The enzymes palmitoyl acyltransferases are necessary for palmitoylation, and the acyl protein thioesterases are required for the removal of palmitoyl residues. This process affects several parts of the target proteins. Palmitoylation facilitates the translocation of membrane proteins into raft zones (e.g., flotillins, which form part of the 215 proteins modified by this process).

Zhang et al. pointed out in their research the importance of sphingosine-1-phosphate in thrombopoiesis investigated on genetically deficient mice. They concluded that sphingosine-1-phosphate is necessary for the platelet formation from megakaryocytes in the bone marrow and their release in the blood, i.e., directional migration of proplatelet-containing cytoplasmic extensions into the circulatory compartment and the shedding of proplatelets in a Rac-dependent manner [22]. Platelet aggregation is highly dependent on the platelet membrane cholesterol content and increases with increasing cholesterol concentration, but the mechanism remains to be clarified. Many signal transduction events are increased following cholesterol enrichment of platelets. These events include increased release of arachidonic acid, indicative of phospholipase A2 activity [1,23].

## 2. Antiplatelet Therapy

Numerous studies have examined and determined the mechanisms of platelet function and their role in the pathogenesis of cardiovascular diseases, which has enabled the discovery and application of a large number of different agents that act effectively and purposefully in the prevention and treatment of these diseases.

The central role in the initiation of blood coagulation belongs to the Von Willebrand factor (VWF), a multimeric plasma glycoprotein constitutively produced by megakaryocytes (i.e., platelet α-granules), endothelium, and subendothelial connective tissue [24]. VWF maintains primary hemostasis through interactions between its several functional domains, coagulation Factor VIII, the collagen beneath endothelial cells exposed by the blood vessels damage, and platelet GPIbα receptors [24,25]. In this way, VWF delivers both platelets and Factor VIII to vascular injury sites, promoting platelet adhesion and consequent platelet plug formation [26]. Plasma VWF levels vary widely across the healthy human population [27]. While individuals with low VWF levels are more prone to mucocutaneous bleeding, individuals with high VWF levels are at higher risk for venous thromboembolic disease, coronary artery disease, and stroke [28].

Platelet agonists such as adenosine diphosphate and/or epinephrine also represent an important thrombogenic factor. It is known that their low concentration is strongly correlated with platelet hyperaggregability, a hemostatic disorder referred to as sticky platelet syndrome (SPS) [29]. Several genes have been associated with the SPS pathogenesis [30]. Analysis of 7 of these genes in patients with deep vein thrombosis (i.e., platelet endothelial aggregation receptor 1 (PEAR1), murine retrovirus integration site 1 (MRVI1), janus kinase 2 (JAK2), FCER1G, proplatelet basic protein (PPBP), alpha2A adrenergic receptor (ADRA2A), and sonic hedgehog (SHH)) supported the idea that genetic variability of PEAR1 and ADRA2A genes are associated with venous thromboembolism [29].

Under physiological conditions, platelets do not adhere to the walls of blood vessels, nor are they activated by the vascular endothelium. However, when atherosclerotic plaques are present in the vascular system, they are subject to sudden disruption (rupture, erosion, or fissure), a signal for platelet activation and thrombus formation [31,32]. The release of thromboxane A2 and adenosine diphosphate (ADP) causes further enhancement of the process and stimulation of a larger number of platelets. As P2Y12 inhibitors have other effects than antiplatelet, the question is whether discontinuation of these drugs may be a signal for activation of proinflammatory and proaggregatory markers, especially in patients with dyslipidemia.

Antiaggregating therapy involves agents that aim to reduce/slow down platelet aggregation and prevent thrombus formation, which is also called antiplatelet treatment. Antiplatelet drugs are much more effective in treating arterial thrombosis, consisting primarily of platelets instead of anticoagulant medications, which are more effective in treating venous thrombosis. Different classes of antiplatelet drugs act at different levels, so we distinguish between drugs that inhibit the enzyme cyclooxygenase, drugs that increase the level of platelet cyclic adenosine monophosphate (cAMP), platelet phosphodiesterase inhibitors, inhibitors of thromboxane synthetase receptor inhibitors, antagonists of thromboxane synthetase, thromboxane A2 receptor antagonists, synthesis inhibitors and thromboxane A2 receptor blockers, adenosine diphosphate (ADP) signal inhibitors, fibrinogen receptor inhibitors or antagonists and others.

Dual antiplatelet therapy aimed at preventing atherothrombotic outcomes has become the standard and indispensable part of treatment in patients with various clinical forms of ischemic heart disease undergoing percutaneous coronary intervention (PCI) [33,34]. Representatives of each of the groups of antiplatelet drugs have been clinically tested. However, it has been proven that cyclooxygenase inhibitors (aspirin) and ADP antagonists (platelet P2Y12 inhibitors) have the highest efficiency and application. Since ADP and thromboxane A2 are the most important platelet agonists released at the thrombus formation site, combined antiplatelet therapy of P2Y12 inhibitors—drugs that block ADP-dependent platelet aggregation and aspirin—that irreversibly inhibit the enzyme cyclooxygenase (thus the thromboxane A2) is more effective than aspirin alone or a combination of aspirin and anticoagulant therapy.

Aspirin irreversibly inhibits the enzyme cyclooxygenase in platelets by acetylating the serine component of the active part of the molecule. In that way, the synthesis of prostaglandin peroxides (PGG2 and PGH2), from which thromboxane A2 is synthesized in platelets, is disabled. Thromboxane A2 causes platelet activation, vasoconstriction of blood vessels, and stimulates atherogenesis by inducing the proliferation of vascular smooth muscle cells. Inhibition of cyclooxygenase is fast, achieved in small doses, irreversible, and lasts the entire lifespan of platelets since platelets are cells without a nucleus and cannot create new proteins [35,36].

P2Y12 receptor blockers are drugs that bind to the P2Y12 class of platelet ADP receptors, thereby blocking the aggregating effect of ADP. ADP binds adjacent platelets through P2Y purinergic receptors bound to the G protein. There are two subtypes of P2Y receptors on the platelet membrane, one of which is attached to Gq and the other to Gi protein. ADP via P2Y1 receptors bound to Gq protein leads to phospholipase C activation, induces a short-term increase in calcium in the cytosol, and leads to transient (short-term) platelet aggregation [37]. In contrast, activation of P2Y12 receptors bound to Gi protein releases Gi protein subunits of αGi and βγ, which through independent signaling events lead to prolonged platelet aggregation [38]. Through the αGi subunit, the cAMP level is reduced by inhibition of the enzyme adenylate cyclase. This decrease in cAMP leads to reduced activation of a specific protein kinase, which causes a lack of phosphorylation of vasodilator-stimulated phosphoprotein (VASP), a crucial event for GPIIb/IIIa receptor inhibition. The βγ subunit activates phosphatidylinositol-3 kinase, an important signaling molecule for P2Y12-mediated secretion of dense granules and GPIIb/IIIa receptor activation [39]. Signaling events downstream of the P2Y12 receptor mediates thromboxane A2 production, α granule release, and P selectin’s subsequent expression on platelet activation [40]. The P2Y12 receptor is also involved in platelet growth and stabilization [41]. The P2Y12 inhibitors include ticlopidine, clopidogrel, prasugrel, ticagrelor, and cangrelor, but clopidogrel, prasugrel, and ticagrelor are mainly used.

The choice, initiation, and duration of dual antiplatelet therapy depend on the clinical presentation of the disease (elective, acute, or emergency intervention) and the type of stent implanted. To achieve the expected therapeutic efficacy and the simultaneous reduction in the risk of bleeding and ischemic risks, an individual approach to each patient is necessary. Following current recommendations, in patients with acute coronary syndrome undergoing PCI treated with aspirin, prasugrel or ticagrelor are advised rather than clopidogrel, due to their clinical superiority, where prasugrel should be considered in preference to ticagrelor for patients with acute coronary syndromes without ST-segment elevation (NSTE-ACS) who proceed to PCI. However, clopidogrel remains the drug of choice in stable CAD patients undergoing coronary stent implantation [42,43,44,45,46,47]. The basic characteristics of oral P2Y12 blockers are given in Table 2.

The platelet membrane is rich in cholesterol. It has been shown that ticagrelor can lead to redistribution/reduction in cholesterol content in raft fractions and that ADP can reverse this effect of ticagrelor [51]. On the other hand, ticagrelor and its active metabolite deshydroxyethoxy ticagrelor may increase the rigidity of the entire platelet plasma membrane, which is due to a rise in cholesterol and phosphatidylcholine ether content [6].

It should be noted that every antiplatelet therapy is highly challenging in patients with inherited bleeding disorders, such as hemophilia A and B, or Von Willebrand’s disease [26], the most common bleeding disorder caused by the qualitative or quantitative deficiency of the pro-VWF [52]. Therefore, the risk of bleeding could complicate reperfusion therapy in these patients. As there are no official recommendations or significant randomized studies that have addressed this issue, the treatment of patients with inherited blood diseases and acute coronary syndrome would require an individualized approach and close cooperation between cardiologists and hematologists.

## 3. High-Density Lipoprotein (HDL) and Discontinuation of P2Y12 Inhibitors

High-density lipoproteins represent a heterogeneous group of molecules consisting of apoprotein I or a combination of apoprotein I and II or apoprotein E, phospholipids, sphingomyelin, cholesteryl-esters, and small amounts of triglycerides and unesterified cholesterol. High-density lipoproteins have a protective effect against atherothrombosis [53,54]. HDL is involved in several regulatory mechanisms such as cholesterol efflux capacity (transfer of cholesterol from extrahepatic tissues to the liver for additional metabolism and excretion), antioxidant, antithrombotic, anti-inflammatory, and antiapoptotic activity [55,56]. Additionally, HDL has been recognized as a significant independent predictor of platelet thrombus formation [57].

HDL might have a particular and intriguing role in the enhanced platelet reactivity after cessation of P2Y12 receptor antagonists. After percutaneous coronary interventions (PCI), patients should be treated with aspirin and P2Y12 receptor antagonists for one month to several years of such therapy depending on ischemic and hemorrhagic risk [41,43,45]. After acute coronary syndrome, most patients stop P2Y12 receptor antagonists 12 months after the PCI, and the increase in ischemic events was confirmed in some [58,59,60], but not all studies [61]. Our research shows that increased blood levels of soluble CD40 ligand (soluble CD40L) and high-sensitivity CRP (hsCRP) are associated with serum HDL concentration in patients treated PCI who used a combination of aspirin and clopidogrel for one year after stopping P2Y12 antagonist. Namely, patients with the lowest serum levels of HDL had the highest increase in soluble CD40L and hsCRP in their blood in the weeks after cessation of clopidogrel. Other blood markers of lipid and glucose status did not correlate to the increase in soluble CD40L and hsCRP after clopidogrel cessation [62]. This association of HDL and soluble CD40L and hsCRP was presented for at least 45 days after the end of clopidogrel therapy and might be a marker of higher risk for coronary thrombotic events, late stent thrombosis, or thrombosis at the new site in the coronary tree.

One explanation for the effect of HDL on prothrombotic signal induction and platelet reactivity includes a direct impact on platelets through their receptors. Namely, Scavenger receptors (SR) are expressed on the surface of platelets, and these receptors are characterized by the ability to recognize native or oxidized forms of various lipoproteins. Receptor classes SR-BI and CD36 have the largest role in lipid/lipoprotein metabolism [63]. CD36 (cluster of differentiation 36; glycoprotein IV or glycoprotein IIIb) receptors are present on the membranes of many cells, including platelets. They play a key role in the turnover of calcium, leukotrienes, fatty acids, and the exchange of cellular signals. Their deficiency is associated with many signs of COVID-19, including increased platelet aggregation that accompanies increased levels of arachidonic acid in the serum of COVID-19 patients [64,65]. Congenital CD36 deficiency also exists in alloimmune thrombocytopenia. However, the links between serum lipids, CD36 receptor regulation, and platelet aggregability are complex and need to be elucidated. For example, CD36 has been linked to oxidized lipid stress (i.e., oxidized low-density lipoproteins, oxLDL associated with dyslipidemia) to platelet hyperactivity [66]. Namely, oxLDL can increase the hydrolysis of cAMP in platelets by leading to phosphorylation and activation of phosphodiesterase 3A (PDE3A) in the presence of Src family kinases, Syk tyrosine kinase, and protein kinase C. Finally, hyposensitivity to prostacyclin (PGI2) occurs. However, this does not directly explain how lowered HDL levels can increase platelet reactivity (which we observed in cardiac patients, see above), although CD36s are scavenging receptors for HDL uptake [67].

The type and degree of HDL oxidation determine the affinity for the corresponding receptor—SR-BI or CD36—and thus their ability to stimulate or inhibit platelet function. Scavenger receptors B type I (SR-BI) are the principal HDL receptors. Their expression is positively correlated with HDL levels, and the inhibitory effect of native and moderately oxidized HDL and SR-BI ligands is absent in the case of SR-BI-deficient platelets [68,69]. SR-BI are present on the surface of endothelial cells. HDL binding to endothelial SR-BI provokes the formation of nitric oxide (NO) by up-regulating endothelial NO synthase [70], which has an additional protective, anti-inflammatory effect on blood vessels.

The association between low HDL and increased platelet aggregability can be indirectly explained by HDL increasing uptake of oxLDL in inflammatory adipocytes via PPARγ/CD36 Pathway [71]. Thus, in HDL deficiency, more oxLDL reaches platelets, so the described mechanism increases their activation. On the other hand, highly oxidized HDL via the CD36 receptor appears to cause a prothrombotic and proinflammatory effect in a dose-dependent manner, and the blockade of the binding of oxidized HDL to CD36 weakens platelet stimulation [72].

It is essential to point out that people with low HDL levels have significantly increased values of oxidized choline glycerophospholipids (such as oxPCCD36), which is a high-affinity ligand for CD36 [73], which also affirms platelet activation. These theses can be confirmed by the fact that in patients with lower HDL levels, after abolishing clopidogrel and the cessation of its antithrombotic effect, more significant changes in inflammatory markers are released from activated platelets are observed [62]. It is unknown whether this association with platelet activation and inflammatory response is unique for the clopidogrel cessation or it is a class effect of P2Y12 receptor antagonists.

Aspirin and clopidogrel work synergistically to control platelet and inflammatory response in patients with coronary disease. The cessation of clopidogrel also influenced aspirin response on platelet function [74]. Aspirin could increase SR-BI [75], a ligand for HDL. Therefore, patients with low HDL levels might be more prone to platelet hyper-reactivity in the period after clopidogrel cessation.

If we keep in mind that cardiovascular diseases are the diseases with the highest morbidity and mortality rate, it is clear that a large number of patients need dual antiplatelet therapy as the first line of defense. Therefore, further research is required to assess whether the loss of P2Y12 receptor blockade represents a significant prothrombotic and proinflammatory impulse, especially in patients at increased cardiovascular risk, such as those with low HDL. It is also necessary to extend the research to other P2Y12 antagonists, such as prasugrel and ticagrelor, since previous studies have focused mostly on clopidogrel.

## 4. Future Perspectives

The development of new analytical techniques opens the field of lipidomics to study factors that affect platelet function and the action of antiplatelet drugs [1]. Over the last two decades, sophisticated methods such as mass liquid chromatography-mass spectrometry (LC/MS) and tandem splicing (ESI) coupled to the tandem (triple quadrupole or MS/ MS) have entered research practice. Thus, it became possible to simultaneously monitor changes in the entire lipidome, i.e., lipid signals in physiological and pathological conditions. Such changes are specific and make it possible to find new drugs’ action targets. In a series of experiments in models of ischemia-reperfusion injury of the kidney and liver, we showed that the lipid profile differs depending on the tissue and that drugs that modulate lipid metabolism in mitochondria and peroxisomes have different effects on that profile [76,77]. Lipidomics became even more important because lipid mediators cannot be deposited in vesicles but are created “on-demand” at the site of action in the cell. Another possibility is the transport of lipid signals between cells via exosomes [78].

It is known that platelet-derived extracellular vesicles can play the role of intercellular signals. It has recently been confirmed that extracellular vesicles are enriched in the fatty acid precursors of lipid mediators and that in humans, they can participate in the regulation of inflammation and healing [79].

## Figures and Tables

**Table 1:** Lipid classification based on Lipid Maps Structure Database.

Lipid Categories	
**01. Fatty Acyls (FA)**	**04. Sphingolipids (SP)**
(FA01) Fatty Acids and Conjugates	(SP01) Sphingoid bases
(FA02) Octadecanoids	(SP02) Ceramides
(FA03) Eicosanoids	(SP03) Phosphosphingolipids
(FA04) Docosanoids	(SP04) Phosphonosphingolipids
(FA05) Fatty alcohols	(SP05) Neutral glycosphingolipids
(FA06) Fatty aldehydes	(SP06) Acidic glycosphingolipids
(FA07) Fatty esters	(SP07) Basic glycosphingolipids
(FA08) Fatty amides	(SP08) Amphoteric glycosphingolipids
(FA09) Fatty nitriles	(SP09) Arsenosphingolipids
(FA10) Fatty ethers	(SP00) Other Sphingolipids
(FA11) Hydrocarbons	**05. Sterol Lipids (ST)**
(FA12) Oxygenated hydrocarbons	(ST01) Sterols
(FA13) Fatty acyl glycosides	(ST02) Steroids
(FA00) Other Fatty Acyls	(ST03) Secosteroids
**02. Glycerolipids (GL)**	(ST04) Bile acids and derivatives
(GL01) Monoradylglycerols	(ST05) Steroid conjugates
(GL02) Diradylglycerols	(ST00) Other Sterol lipids
(GL03) Triradylglycerols	**06. Prenol Lipids (PR)**
(GL04) Glycosylmonoradylglycerols	(PR01) Isoprenoids
(GL05) Glycosyldiradylglycerols	(PR02) Quinones and hydroquinones
(GL00) Other Glycerolipids	(PR03) Polyprenols
**03. Glycerophospholipids (GP)**	(PR04) Hopanoids
(GP01) Glycerophosphocholines	(PR00) Other Prenol lipids
(GP02) Glycerophosphoethanolamines	**07. Saccharolipids (SL)**
(GP03) Glycerophosphoserines	(SL01) Acylaminosugars
(GP04) Glycerophosphoglycerols	(SL02) Acylaminosugar glycans
(GP05) Glycerophosphoglycerophosphates	(SL03) Acyltrehaloses
(GP06) Glycerophosphoinositols	(SL04) Acyltrehalose glycans
(GP07) Glycerophosphoinositol monophosphates	(SL05) Other acyl sugars
(GP08) Glycerophosphoinositol bisphosphates	(SL00) Other Saccharolipids
(GP09) Glycerophosphoinositol trisphosphates	**08. Polyketides (PK)**
(GP10) Glycerophosphates	(PK01) Linear polyketides
(GP11) Glyceropyrophosphates	(PK02) Halogenated acetogenins
(GP12) Glycerophosphoglycerophosphoglycerols	(PK03) Annonaceae acetogenins
(GP13) CDP-Glycerols	(PK04) Macrolides and lactone polyketides
(GP14) Glycosylglycerophospholipids	(PK05) Ansamycins and related polyketides
(GP15) Glycerophosphoinositolglycans	(PK06) Polyenes
(GP16) Glycerophosphonocholines	(PK07) Linear tetracyclines
(GP17) Glycerophosphonoethanolamines	(PK08) Angucyclines
(GP18) Di-glycerol tetraether phospholipids	(PK09) Polyether antibiotics
(GP19) Glycerol-nonitol tetraether phospholipids	(PK10) Aflatoxins and related substances
(GP20) Oxidized glycerophospholipids	(PK11) Cytochalasins
(GP00) Other Glycerophospholipids	(PK12) Flavonoids
	(PK13) Aromatic polyketides
	(PK14) Non-ribosomal peptide/polyketide hybrids
	(PK15) Phenolic lipids
	(PK00) Other Polyketides

**Table 2:** Characteristics of oral P2Y12 inhibitors [46,47,48,49,50].

	Ticlopidine	Clopidogrel	Prasugrel	Ticagrelor
Chemical structure	Thienopyridine	Thienopyridine	Thienopyridine	Cyclopentyltri azolopyrimidine
Receptor binding	Irreversible	Irreversible	Irreversible	Reversible
Prodrug	Yes	Yes	Yes	No
Onset of action	~6 h	2–8 h	0.5–4 h	0.5–4 h
Half-life (active metabolite)	12.6 h	30 min	7 h	9 h
Metabolism	Primarily CYP2B6 and CYP2C19	Primarily CYP2C19	Primarily hydrolysis by esterases	Primarily CYP3A4
Loading dose	500 mg	300 or 600 mg	60 mg	180 mg
Maintenance dose	2 × 250 mg	75 mg	10 mg	2 × 90 mg
Cessation before non-emergent surgery	At least fivedays	At least five days	At least seven days	At least three days
Elimination	Urine 60% and feces 23%	Urine 50% and feces 46%	Urine 68% and feces 27%	Urine 26% and feces 58%
